# Molecular techniques for sex identification of captive birds

**DOI:** 10.14202/vetworld.2019.1506-1513

**Published:** 2019-09

**Authors:** Medania Purwaningrum, Herjuno Ari Nugroho, Machmud Asvan, Karyanti Karyanti, Bertha Alviyanto, Randy Kusuma, Aris Haryanto

**Affiliations:** 1 Department of Biochemistry and Molecular Biology, Faculty of Veterinary Medicine, Universitas Gadjah Mada, Yogyakarta 55281, Indonesia; 2 Research Centre for Biology, Indonesian Institute of Sciences, Jl Jakarta-Bogor Km. 46, Cibinong, West Java 16911, Indonesia; 3 Gembira Loka Zoo and Botanical Garden, Yogyakarta 55171, Indonesia

**Keywords:** bird, chromodomain helicase DNA-binding gene, molecular bird sexing, polymerase chain reaction, sexing

## Abstract

**Background and Aim:**

Many avian species are considered sexually monomorphic. In monomorphic bird species, especially in young birds, sex is difficult to identify based on an analysis of their external morphology. Accurate sex identification is essential for avian captive breeding and evolutionary studies. Methods with varying degrees of invasiveness such as vent sexing, laparoscopic surgery, steroid sexing, and chromosome inspection (karyotyping) are used for sex identification in monomorphic birds. This study aimed to assess the utility of a non-invasive molecular marker for gender identification in a variety of captive monomorphic birds, as a strategy for conservation.

**Materials and Methods:**

DNA was isolated from feather samples from 52 individuals representing 16 species of 11 families indigenous to both Indonesia and elsewhere. We amplified the chromodomain helicase DNA-binding (CHD) gene using polymerase chain reaction with MP, NP, and PF primers to amplify introns with lengths that differ between the CHD-W and the CHD-Z genes, allowing sex discrimination because the W chromosome is exclusively present in females.

**Results:**

Molecular bird sexing confirmed 33 females and 19 males with 100% accuracy. We used sequencing followed by alignment on one protected bird species (*Probosciger aterrimus*).

**Conclusion:**

Sex identification may be accomplished noninvasively in birds, because males only have Z sex chromosomes, whereas females have both Z and W chromosomes. Consequently, the presence of a W-unique DNA sequence identifies an individual as female. Sexing of birds is vital for scientific research, and to increase the success rate of conservation breeding programs.

## Introduction

Birds are one of Indonesia’s major biological assets. Of the approximately 10,000 bird species in the world, 1598 are found in Indonesia, of which approximately 372 are endemic [1]. Sex determination is essential for captive breeding of birds, but is among several obstacles to success in this endeavor. Approximately 60% of bird species are monomorphic [2], with sex identification of both young and mature individuals based only on morphological analysis of the phenotype. Sex identification in such species requires vent sexing, laparoscopy, steroid sexing, or karyotyping [3], or by comparing blood plasma protein profiles between male and female individuals [4]. The feasibility of these methods depends on laboratory facilities and researcher expertise. Vent sexing requires a truly skilled person. Laparoscopy is high-risk, especially when applied to small birds because it requires surgery followed by intensive post-operative care. Steroid hormone concentrations differ significantly between the egg yolks of male and female [5], but the application of such analyses for sex identification requires further research, especially on the accuracy and specifications of hormone measurements [6].

Avian sex chromosomes differ from those of mammals, in which male sex chromosomes are heterozygous (XY) and females are homozygous (XX). Sex chromosomes of birds are the opposite: Females have heterozygous (ZW) and males homozygous (ZZ) sex chromosomes [7]. Two decades ago, sex identification in ratites was accomplished using size differences in introns of the chromodomain helicase DNA-binding (CHD) gene between the Z and W chromosomes [8]. Amplification of the CHD gene segment in male birds only produces one amplicon fragment of the Z chromosome, while in females it produces two fragments of the Z and W chromosomes that differ in ribbon length due to length differences between the amplified introns. Although relatively expensive, sex identification using molecular methods can be applied to young birds and monomorphic birds and has high accuracy because it targets the sex chromosomes directly. Chromosome characterization using karyotyping compares the size of the W chromosome to the larger Z chromosome, but the difficulty of obtaining good cell cultures reduces the utility of this method [9]. More recently, the introduction of sex identification using molecular techniques [10] has reduced reliance on non-molecular techniques and opened new opportunities for researchers interested in avian sex identification [11]. Molecular techniques expedite identification of a bird’s sex because they can be applied in birds as young as 5-7 days, and take only 1 day to complete. In contrast, non-molecular bird sexing can only be accomplished once the birds reach adulthood.

The variety of bird sexing methods currently in use have several weaknesses: Karyotyping and steroid hormone analysis are time-consuming and relatively costly, laparotomy and laparoscopy are invasive and challenging to apply in the field, cloacal examination and observations of mating behavior can only be used during particular seasons. Other methods of determining sex include morphometric analysis [12] and molecular approaches [13] including amplification of the CHD-1 gene segment, and other sites on the sex chromosome. Amplification can be achieved with conventional polymerase chain reaction (PCR) and also using microsatellite amplification, random amplified polymorphism DNA, restriction fragment length polymorphism, or real-time PCR. The advantages of the conventional PCR that we used are its relative ease of application and low cost [14].

Male and female characteristics of monomorphic birds in Indonesia usually involve dimensions of body shape, body weight, head shape, and tail feathers in adult birds [15]. Bird sex can be ascertained from the CHD-W gene using the Po primer and multiplex PCR methods [16], and real-time PCR [14]. Sex identification of birds for breeding purposes may use the P8/P2 primer on the CHD gene for the two sex chromosomes, W and Z [17]. Three primers: HD1F/CHD1R, 2550F/2718R, and P2/P8 are used to determine the sex of birds [18]; PCR methods are more accurate than morphometry and DNA finger-printing for sex identification in birds [19]. Molecular methods are more accurate and have higher sensitivity than morphometry when sample sizes are small [13]. Gender differentiation and age estimation can be accomplished using morphology. Molecular identification of bird sex usually requires blood samples [20], or tissue samples with the amplification refractory mutation system using the primers P2, NP, and MP [21]. Non-invasive sex identification using tissue originating from the feather calamus has been carried out using primers P2 and P8, which yield a PCR product of 350 bp for the Z chromosome and 400 bp for the W chromosome [22]; primers 2550F and 2718R have also been used with this tissue [23-25].

This study aimed to differentiate bird sex using molecular sexing, with the ultimate aim of assisting with breeding, conservation, and ecological processes in protected and rare birds.

## Materials and Methods

### Ethical approval

This study met the ethical requirements of both the Ethical Clearance Commission of the Faculty of Veterinary Medicine, Gadjah Mada University (Approval no. 0013/EC-FKH/Int./2018), and local laws and regulations.

### Specimen collection

We used a total of 48 bird feather samples from the Gembira Loka Yogyakarta Zoo, and six from the Breeding Facility of the Biology Research Centre, Indonesian Institute of Sciences (Lembaga Ilmu Pengetahuan Indonesia [LIPI]) (Table-1) [26-43]. All samples were stored at 37°C without using preservatives until DNA isolation.

**Table 1:** Complete list of bird species, name, number, and conservation status for all feather samples collected from both the Gembira Loka Zoo and LIPI [26].

Family	Scientific name	Common name	Quantity	Conservation status
*Psittacidae*	*Eclectus roratus*	Roratus parrot	2	Protected by P. 92/2018 [27] IUCN: Least Concern [28]
*Cacatuidae*	*Cacatua sanguinea*	Little corella	2	Protected by P. 92/2018 [27] IUCN: Least Concern [29]
*Cacatuidae*	*Probosciger aterrimus*	Palm/Great black cockatoo	6	Protected by P. 92/2018 [27] IUCN: Least Concern [30]
*Psittaculidae*	*Lorius lory*	Black-capped lory	4	Protected by P. 92/2018 [27] IUCN: Least Concern [31]
*Psittrichasiidae*	*Psittrichas fulgidus*	Pesquet’s/Vulturine parrot	3	Protected by P. 92/2018 [27] IUCN: Vulnerable [32]
*Pelecanidae*	*Pelecanus onocrotalus*	Great white pelican	2	Protected by P. 92/2018 [27] IUCN: Least Concern [33]
*Anatidae*	*Cygnus atratus*	Black swan	4	IUCN: Least Concern [34]
*Phoenicopteridae*	*Phoenicoparrus minor*	Lesser flamingo	6	IUCN: Near Threatened [35]
*Psittacidae*	*Ara ararauna*	Blue-and-yellow macaw	5	IUCN: Least Concern [36]
*Cacatuidae*	*Eolophus roseicapilla*	Galah cockatoo	2	IUCN: Least Concern [37]
*Spheniscidae*	*Spheniscus demersus*	Jackass/African penguin	2	IUCN: Endangered [38]
*Psittacidae*	*Psittacus erithacus*	African gray parrot	6	IUCN: Endangered [39]
*Psittacidae*	*Amazona amazonica*	Orange-winged Amazon	3	IUCN: Least Concern [40]
*Psittacidae*	*Aratinga solstitialis*	Sun conure/Sun parakeet	2	IUCN: Endangered [41]
*Threskiornithidae*	*Eudocimus ruber*	Scarlet ibis	4	IUCN: Least Concern [42]
*Musophagidae*	*Tauraco persa*	Green turaco	1	IUCN: Least Concern [43]

LIPI=Lembaga Ilmu Pengetahuan Indonesia

### DNA extraction

A commercial gSYNC DNA extraction kit was used according to manufacturer’s instructions, with some modification. Briefly, a 0.5-1 cm piece from at least three feathers, including the calamus of each sample (n=54) was cut and transferred into 1.5 ml Eppendorf tube. 200 µl of GST Buffer and 20 µl of Proteinase K were then added to each sample tube, mixed by micropestle, and incubated overnight at 60°C while shaken every 5 min. 200 µl GSB Buffer was then added to each sample tube, mixed vigorously, and incubated at 60°C for 20 min while shaken every 5 min. Procedures were then continued according to the gSYNC DNA Extraction Kit instructions.

200 µl of absolute ethanol was added to each tube, mixed immediately for 10 s; then the tube contents were transferred to the GS column in a 2 ml collection tube. All supernatant was transferred into the GS column that had been fitted with a collection tube and then centrifuged at a speed of 15,000× *g* for 1 min. The solution in the collection tube was discarded. For washing, 400 µl of W1 buffer was added to the GS column of each sample, which was then centrifuged at 15,000× *g* for 30 s. The solution remaining in the collection tube was then discarded, 600 µl of wash buffer added to each GS column, and the column then centrifuged at 15,000× *g* for 30 s. The solution remaining in the collection tube was again discarded, and the GS column dried by centrifuging again at 15000× *g* for 3 min.

The dried GS column was transferred to a clean, dry 1.5 ml microcentrifuge tube, and 75 µl of elution buffer that had been incubated at 60°C added. The tube was then centrifuged at 15,000× *g* for 30 s, and 25 µl of elution buffer added before another centrifugation at 15,000× *g* for 30 s to ensure that all DNA in the GS column was filtered properly.

### PCR

DNA was extracted from bird feather samples collected at the Yogyakarta Gembira Loka Zoo and Captive Facility of the LIPI Biology Research Centre using the gSYNC DNA extraction kit. Extracted DNA was stored at −20°C/−80°C until it was used. DNA extraction product can be directly amplified by PCR: DNA fragments were amplified by targeting the CHD gene on the sex chromosome DNA using P2, NP, and MP primers. The base composition of the primers, annealing temperature (Tm), and melting Tm is presented in Table-2 [44].

**Table 2:** Base composition and melting Tm for P2, NP, and MP primers used in CHD gene amplification [44].

Primer	Nucleotide structure	Number of base	Temperature (°C)
NP	5′-GAGAAACTGTGCAAAACAG-3′	20	49.5
P2	5′-TCTGCATCGCTAAATCCTTT-3′	19	51.9
MP	5′-AGTCACTATCAGATCCGGAA-3′	20	52.3

Tm=Temperature, CHD=Chromodomain helicase DNA-binding

A mixture of PCR components for bird DNA in one reaction with a total volume of 25 µl consisting of MyTaq^™^ DNA Polymerase, forward primer (10 pmol), reverse primer (10 pmol), ddH_2_O, and isolated DNA, was used as a template (Table-3).

**Table 3:** The composition of reagent mixture of PCR DNA in a sample reaction for the CHD gene.

MyTaq™ DNA polymerase (μl)	Primer forward PF (μl)	Primer reverse NP (μl)	Primer reverse MP (μl)	Total DNA (μl)	Total (μl)
12.5	1	1	1	9.5	25

CHD=Chromodomain helicase DNA-binding, PCR=Polymerase chain reaction

PCR amplification comprised three stages: Denaturation at 94°C for 20 s, annealing at 46°C for 30 s, and extension at 72°C for 40 s. The stages of PCR amplification were repeated for 40 cycles. The PCR process began with pre-denaturation at 94°C for 2 min and ended with final extension at 72°C for 10 min. As the positive control, isolated DNA from female and male monomorphic birds of known sex was used.

### DNA electrophoresis

**Figure-1:**
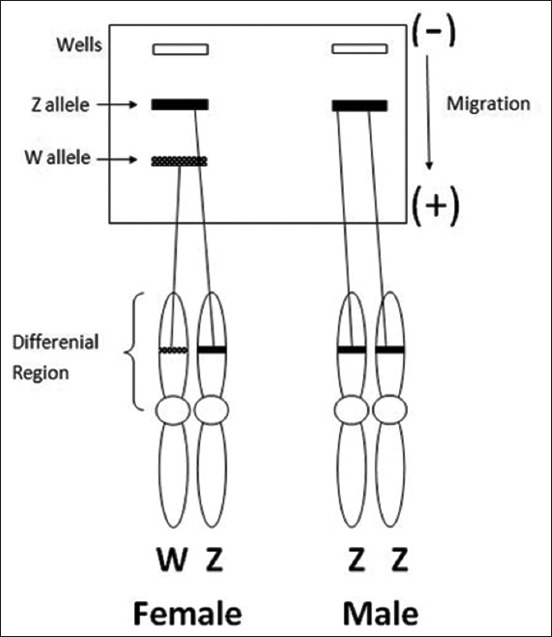
Scheme of DNA amplification targets on W and Z sex chromosomes of male and female birds.

DNA electrophoresis was carried out on 2.5% agarose gel with FluoroSafe staining in 100 ml of 1× Tris/Borate/EDTA buffer solution. PCR results from male and female bird feather samples were expected to show very different results, with a single DNA band in males and two in females (representative schematic in Figure-1).

## Results

**Figure-2:**
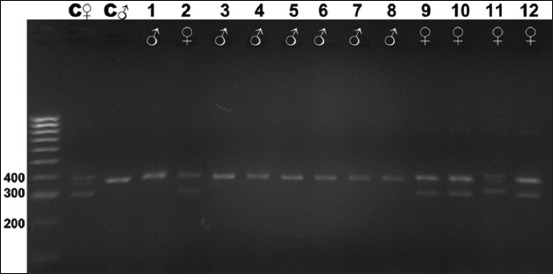
Electrophoresis gel showing polymerase chain reaction product for the chromodomain helicase DNA-binding genes of birds captive at the KRKB Gembira Loka Yogyakarta: The roratus parrot *Eclectus roratus*, little corella *Cacatua sanguinea*, palm/great black cockatoo *Probosciger aterrimus*, and black-capped lory *Lorius lory*. M: Marker/ladder DNA, 100-1000 bp; C ♀: Female control roratus parrot; C ♂: Male control roratus parrot; 1-2: Little corella, (1 male/1 female); 3-8: Palm/great black cockatoo (6/0); 9-12: Black-capped lory (0/4).

**Figure-3:**
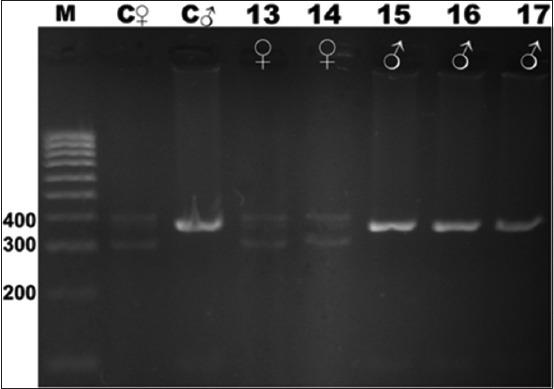
Electrophoresis gel showing polymerase chain reaction product for the chromodomain helicase DNA-binding genes of birds captive at the KRKB Gembira Loka Yogyakarta: Pesquet’s/vulturine parrot *Psittrichas fulgidus*, and great white pelican *Pelecanus onocrotalus*. M: Marker/ladder DNA, 100-1000 bp; C ♀: Female control roratus parrot *Eclectus roratus*; C ♂: Male control roratus parrot; samples 13-15: Pesquet’s/vulturine parrot (1 male/2 females); 16-17: great white pelican (2/0).

**Figure-4:**
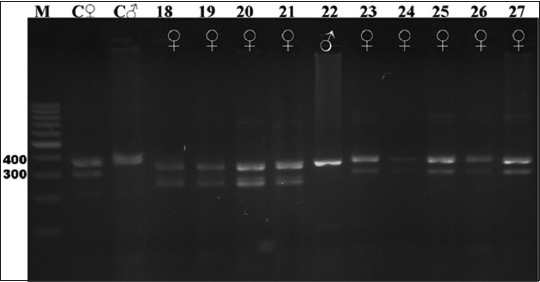
Electrophoresis gel showing polymerase chain reaction product for the chromodomain helicase DNA-binding genes of birds captive at the KRKB Gembira Loka Yogyakarta: Black swan *Cygnus atratus*, and lesser flamingo *Phoenicoparrus minor*. M: Marker/ladder DNA, 100-1000 bp; C ♀: Female control roratus parrot *Eclectus roratus*; C ♂: Male control roratus parrot; samples 18-21: Black swan (0 males/4 females); 22-27: Lesser flamingo (1/5).

**Figure-5:**
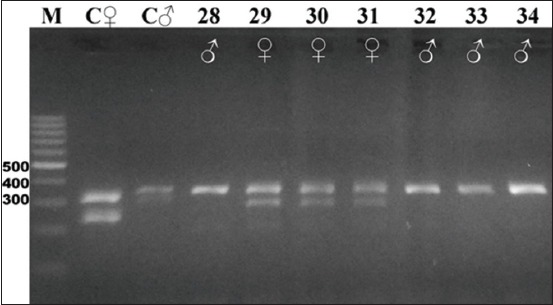
Electrophoresis gel showing polymerase chain reaction product for the chromodomain helicase DNA-binding genes of birds captive at the KRKB Gembira Loka Yogyakarta: Macaw *Ara ararauna*, and galah cockatoo *Eolophus roseicapilla*. M: Marker/ladder DNA, 100-1000 bp; C ♀: Female control roratus parrot *Eclectus roratus*; C ♂: Male control roratus parrot; samples 28-32: Macaw (2 males/3 females); 33-34: Galah cockatoo (2/0).

**Figure-6:**
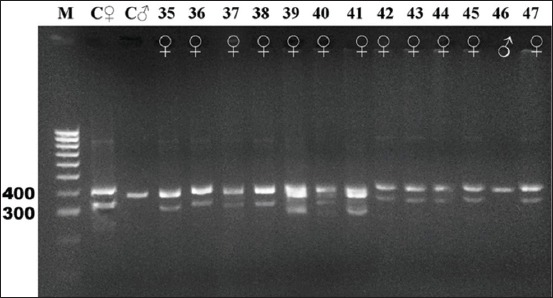
Electrophoresis gel showing polymerase chain reaction product for the chromodomain helicase DNA-binding genes of birds captive at the KRKB Gembira Loka Yogyakarta: Jackass/African penguin *Spheniscus demersus*, African gray parrot *Psittacus erithacus*, orange-winged Amazon cockatoo *Amazona amazonica*, and sun conure *Aratinga solstitialis*. M: Marker/ladder DNA, 100-1000 bp; C ♀: Female control roratus parrot *Eclectus roratus*; C ♂: Male control roratus parrot; samples 35-36: Jackass/African penguin (0 male/2 females); 37-42: AFRICAN gray parrot (0/6); 43-45: Orange-winged Amazon cockatoo (0/3); 46-47: Sun conure (1/1).

**Figure-7:**
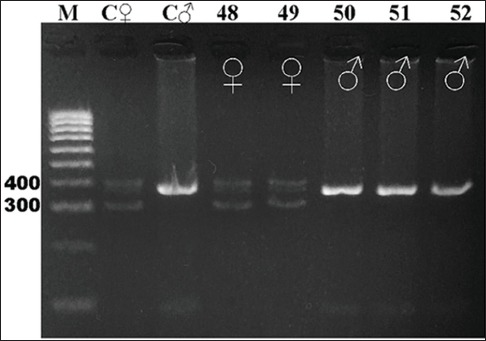
Electrophoresis gel showing polymerase chain reaction product for the chromodomain helicase DNA-binding genes of birds captive at the KRKB Gembira Loka Yogyakarta: Scarlet ibis *Eudocimus ruber*, and green turaco *Tauraco persa*. M: Marker/ladder DNA, 100-1000 bp; C ♂: Female control roratus parrot *Eclectus roratus*; C ♀: Male control roratus parrot; samples 48-51: Scarlet ibis (2 males/2 females); 52: Green turaco (1/0).

Gel electrophoresis results of the CHD gene amplification for all 16 species studied are shown in Figures-2-7. For female and male positive controls, we selected the roratus parrot, which has sexually dimorphic plumage and so was suitable for validation of male and female bands. Males of this species are green, and females are red and blue [45].

## Discussion

Based on the visualization of DNA amplification products from the birds sampled, and the positive control individual male and female roratus parrots, our study demonstrates that the CHD gene segment in birds can be used to differentiate sex reliably. All male birds sampled produced a band of PCR product of 400 bp from the amplification of the CHD-binding 1 (CHD1)-Z gene segment, whereas females will produce two bands of PCR product of approximately 400 bp and 300 bp for the CHD1-Z and CHD1-W gene segments, respectively. The clarity and visibility of the difference between males and females depended on which feather was isolated first. While some samples showed clear DNA band results, others yielded unclear results. This difference was attributable to the number of feather roots (the calamus, containing genetic material) included in the sample. Species for which a greater number of calamuses was obtained showed clearer DNA bands on electrophoresis.

We used samples from 52 individuals of monomorphic bird species. The little corella has a body length of approximately 380 mm. The plumage and crest are white; the eyelids are rather wide and blue. This species has five subspecies: (1) *Cacatua sanguinea transfreta* which occurs in Southern Papua and Papua New Guinea; (2) *C. s. sanguinea* in North-western Australia; (3) *Cacatua sanguinea normantoni* in the Western Cape York Peninsula, Australia; (4) *Cacatua sanguinea westralensis* in Western Australia; and (5) *Cacatua sanguinea gymnopis* in central and Eastern Australia [46,47]. The palm/great black cockatoo has a huge black crest, red cheek spots, and a very long bill that is shorted in females; it occurs in Papua [47,48]. The black-capped lory is endemic to Papua and the surrounding small islands; adults are red on the body, black from the forehead to nape, dark blue around the base of the neck, green on the wings, and dark blue from the chest to the lower abdomen. The top of the tail feathers is red with blue tip, while the under tail coverts are olive-yellow; the cere is gray, the legs dark gray, the iris yellow to orange, and the adult has an orange bill. Sexual identification in *L. lory* is relatively difficult to do through observation alone because this bird is monomorphic; there is no significant difference in morphology, size and color of the body between the two sexes [13], so sex identification by molecular means is necessary. Pesquet’s/vulturine parrot is endemic to the hills and Montane of rainforests in New Guinea and the Papua highlands at an altitude of 100-1800 masl [49]. It is black on the head, back, and tail. The neck is grayish, the abdomen and underwing red. The great white pelican is a very large water bird weighing between 4 and 11 kg with a wingspan of 2.75 m, white with partly black wings and a tail. The sex of this species can be determined from cloacal diameter after breeding, although this method is inaccurate [50]. Molecular sexing of this species is thus a solution. The black swan is sexually monomorphic [51]; we adopted the locus-specific PCR approach based on the CHD1 gene for sex determination of this species. The lesser flamingo is a long-legged social species of the family *Phoenicopteridae* that occurs in the western and eastern hemispheres, although it is more common in the eastern hemisphere. On average, males are significantly larger than females in all age groups, although with substantial overlap in all morphological measurements [52]; behavior does not differ between sexes [53].

Chest feathers of the macaw are yellow, and the wings are blue. This sex of this bird can be identified with a universal primer [54]. The galah cockatoo, also known as the rose-breasted, rose, or pink-and-purple cockatoo, is common and widely distributed and occurs open areas almost throughout mainland Australia [55]. Male and female African penguins are similar in overall appearance, which renders sexual differentiation in the field difficult [56]. Molecular sexing applied to both male and female individuals of *Psittacus erithacus*. Morphological sex identification in birds can be difficult if the animals are young or when there is no appreciable sexual dimorphism. Sex determination can be impossible when few and/or degraded biological material (e.g., feathers, blood traces, and decomposed carcasses) is available [57]. *Psittacidae* exhibit one of the highest population declines due to hunting, habitat fragmentation, and degradation, and therefore their conservation is a priority. Sex differentiation in this group is made difficult by sexual monomorphism, and because traditional sexing methods are traumatic or require extensive protocols that do not work on juveniles. Molecular sexing provides a minimally invasive, effective, and rapid technique to determine the sex of individuals [58]. Male and female sun conures are extremely similar; molecular sexing has been performed on this species [54]. The scarlet ibis cannot be sex-typed using P2-P8 primers [59], so we used other primers for this species. Turaco species are sexually monomorphic [60]. Therefore, molecular sexing was performed.

A summary of sex identifications using the CHD gene PCR from 52 monomorphic bird samples and two dimorphic individual roratus parrots as positive controls is presented in Table-4.

**Table 4:** Summary of sex identifications of birds in this study based on CHD gene PCR products.

Family	Scientific name	Common name	Quantity	Male/Female
*Psittacidae*	*Eclectus roratus*	Roratus parrot	2	1/1
*Cacatuidae*	*Cacatua sanguinea*	Little corella	2	1/1
*Cacatuidae*	*Probosciger aterrimus*	palm/great black cockatoo	6	6/0
*Psittaculidae*	*Lorius lory*	Black-capped lory	4	0/4
*Psittrichasiidae*	*Psittrichas fulgidus*	Pesquet’s/Vulturine parrot	3	1/2
*Pelecanidae*	*Pelecanus onocrotalus*	Great white pelican	2	2/0
*Anatidae*	*Cygnus atratus*	Black swan	4	0/4
*Phoenicopteridae*	*Phoenicoparrus minor*	Lesser flamingo	6	1/5
*Psittacidae*	*Ara ararauna*	Blue-and-yellow macaw	5	2/3
*Cacatuidae*	*Eolophus roseicapilla*	Galah cockatoo	2	2/0
*Spheniscidae*	*Spheniscus demersus*	Jackass/African penguin	2	0/2
*Psittacidae*	*Psittacus erithacus*	African gray parrot	6	0/6
*Psittacidae*	*Amazona amazonica*	Orange-winged Amazon	3	0/3
*Psittacidae*	*Aratinga solstitialis*	Sun conure/parakeet	2	1/1
*Threskiornithidae*	*Eudocimus ruber*	Scarlet ibis	4	2/2
*Musophagidae*	*Tauraco persa*	Green turaco	1	0/1

CHD=Chromodomain helicase DNA-binding, PCR=Polymerase chain reaction

Our PCR analyses show that for 16 species of wild birds at both the Gembira Loka Botanical Gardens and Zoo (Gembira Loka KRKB), and LIPI, individuals of the same sex are kept in one cage. For other species in this study, the analyses indicate that male-female pairs are sharing cages, increasing the probability of breeding. These results will be used as a reference for breeding programs in Gembira Loka KRKB and LIPI, to improve the success of breeding programs to protect both Indonesian and exotic birds.

A total of 45 µl of PCR products from palm/great black cockatoo samples were then purified and sequenced using the Sanger method; DNA sequencing of PCR products was carried out both upstream and downstream for each sample. Sequencing results were combined to form the CHD-Z king parrot gene segment. The sequences of CHD-Z gene segments obtained were compared with the CHD-Z segments available at GenBank from the type specimens of the Goffin’s cockatoo/Tinambar corella (*Cacatua goffiniana*, KT022229.1); (*Cacatua moluccensis*, KR019958.1); rose-ringed parakeet (*Psittacula krameri*, FJ913846.1); white-eyed parakeet (*Psitta leucophthalmus*, KT022230.1); scarlet macaw (*Ara macao*, KF412778.1); and red-crowned amazon (*Amazona viridigenalis*, KR019952.1). The analysis was carried out using Mega X software (https://www.megasoftware.net/). The combination sequence of down-stream and up-stream direction products was 332 nt. The alignment results of the CHD-Z gene sequence are presented in Tables-5 and 6.

**Table 5:** Results of CHD-Z gene alignment for the palm/great black cockatoo, *Probosciger aterrimus.*

Gene	Size	Sequence
CHDZ partial	332 nt	GCAAAACAGGTGTCTCTTGGTTCTGACTGACC TGTACTTTTATCTTGCTGTTGGTTTAGTTAGTT TGTTGGGGGTTGTTGTTGGGTTTTGGTGTGG GGTTTTTTTCCTCCTTTTTTGGACACATATTTTT GACAGGCTGTATAAAACTTACTTATCTTTGTT AATGATGTAGCTTTGAACTACTTACTCTGAC ATTCCAGATCAGCTTTAATGGAAGTGAAGG GAGGCGGAGTAGGAGTAGAAGATACTCTG GATCTGATAGTGACTCCATCTCAGAAAGGA AACGGCCAAAAAAGCGTGGAAGACCACG AACTATTCCTCGAGAAAATATT

CHD=Chromodomain helicase DNA-binding

**Table 6:** Changing nucleotide sites based on reference sequences of *Cacatua goffiniana* (KT022229.1).

Different site position													1	1	1	1	1	1	1	2	2	2	2	2

		1	1	3	4	4	6	8	8	8	8	9	1	1	2	2	6	7	9	2	2	5	5	8

	1	3	5	5	0	1	1	5	6	7	9	4	3	9	1	7	5	1	2	3	9	2	9	0
*Cacatua goffiniana*	T	T	G	C	G	C	G	G	T	G	G	C	C	T	G	A	G	G	C	T	G	A	A	A
*Probosciger aterrimus*	C	.	.	.	.	.	.	.	.	.	.	T	.	.	.	.	.	.	.	.	.	.	.	.
*Cacatua moluccensis*	C	C	.	.	.	.	.	.	.	.	.	.	.	.	.	G	.	.	.	.	.	.	.	.
*Psittacula krameri*	C	.	.	G	C	T	T	C	.	T	.	T	.	C	.	.	A	A	.	C	A	.	.	.
*Psittacara leucophthalmus*	C	.	.	G	C	T	.	.	G	T	T	T	.	C	A	.	.	A	T	.	.	G	C	.
*Ara macao*	C	C	A	G	C	T	.	.	G	T	T	T	T	C	.	.	.	A	T	.	.	.	.	G

Based on multiple alignments with several partial sequences of CHD-Z genes, several types of psittacines available in GenBank show differences in several nucleotide sites. The CHD-Z sequence from *P. aterrimus* has two sites which different from CHD-Z sequences from *C. goffiniana* and *C. moluccensis*. The three cockatoos are older sibling species distributed in Eastern Indonesia. Especially for *P. aterrimus*, the distribution reaches the Northern region of Australia.

## Conclusion

Amplification of the 400 bp segment of the CHD1-Z gene and the 300 bp segment of the CHD1-W gene can be used to distinguish sex in captive birds, as demonstrated by our analyses of 16 diverse bird species from 11 families. Our use of male and female roratus parrots as controls confirms the accuracy and reliability of this method, which has great potential for use in conservation breeding programs.

## Authors’ Contributions

MP and AH planned and designed the study. MP, AH, HAN, MA, KK, BA, and RK collected samples. MP, HAN, and AH carried out the work (helped with DNA extraction, PCR examination, DNA electrophoresis, and performed the bioinformatics data analysis). MP drafted the manuscript; AH translated and revised the manuscript. All authors contributed to this research and read and approved the final manuscript.
